# Impedance spectroscopy of the cell/nanovolcano interface enables optimization for electrophysiology

**DOI:** 10.1038/s41378-023-00533-z

**Published:** 2023-05-16

**Authors:** Nicolas Maïno, Arnaud Bertsch, Philippe Renaud

**Affiliations:** Microsystems laboratory 4, Institute of Electrical and Micro Engineering, Ecole Polytechique Fédérale de Lausanne (EPFL), Lausanne, Switzerland

**Keywords:** Bionanoelectronics, Electrical and electronic engineering

## Abstract

Volcano-shaped microelectrodes have demonstrated superior performance in measuring attenuated intracellular action potentials from cardiomyocyte cultures. However, their application to neuronal cultures has not yet yielded reliable intracellular access. This common pitfall supports a growing consensus in the field that nanostructures need to be pitched to the cell of interest to enable intracellular access. Accordingly, we present a new methodology that enables us to resolve the cell/probe interface noninvasively through impedance spectroscopy. This method measures changes in the seal resistance of single cells in a scalable manner to predict the quality of electrophysiological recordings. In particular, the impact of chemical functionalization and variation of the probe’s geometry can be quantitatively measured. We demonstrate this approach on human embryonic kidney cells and primary rodent neurons. Through systematic optimization, the seal resistance can be increased by as much as 20-fold with chemical functionalization, while different probe geometries demonstrated a lower impact. The method presented is therefore well suited to the study of cell coupling to probes designed for electrophysiology, and it is poised to contribute to elucidate the nature and mechanism of plasma membrane disruption by micro/nanostructures.

## Introduction

The emergence of micro/nanoelectrodes for intracellular recording of the transmembrane potential in the last decade is a significant advancement that is poised to supplant traditional extracellular recordings with planar multielectrode arrays (MEAs). This technology aims to allow the recording of the whole electrophysiological repertoire, not only action potential, while retaining the advantages of MEA, including multisite sensing for high-throughput experiments and long-term monitoring due to reduced cell damage, compared to patch clamp techniques Spira, Huang, Shmoel, and Erez^1^. Nonetheless, the establishment of intracellular access by the probe (i.e., micro-nanoelectrode) is a process that remains elusive and hard to study.

A simple approach consists of measuring the yield of intracellular access as the percentage of probes that register an electrophysiological signal with intracellular features (polarity, timescale and amplitude). However, this assessment method is time-consuming, as most electrogenic cell cultures take time to mature (e.g., 2–3 weeks for primary neurons) and require that intracellular access is a consistent, if rare, event. Alternative methods include conventional patch-clamp and micro/nanostructure characterization of the cell/probe interface Lin, Xie, Osakada, Cui, and Cui^2^; Robinson et al.^3^, optical microscopy Berthing et al.^4^; Braun and Fromherz^5^ and electron microscopy Hai et al.^6^; Santoro et al.^7^; Wrobel et al.^8^. Unfortunately, the patch clamp method suffers from an inherent throughput limitation while microscopy techniques resolve the dimension of the cleft between the cell and probe, an indirect measurement that requires nontrivial sample preparation, and whose spatiotemporal resolution can be limiting.

We hereby present an impedance spectroscopy method that allows optimization of the interface of cells on nanovolcano (NV) electrodes. The impedance spectrum of individual cells cultured on arrays of NVs is measured, and the seal resistance, a critical parameter for intracellular electrophysiology recordings, is assessed. The measured seal resistances were found to correlate well with electrophysiological recording quality. This method is label-free, scalable, harmless for cells and does not necessitate the cell to be electrogenic. The last point is a critical advantage considering the present experimental bottleneck of electrogenic culture maturation. Finally, this method can be implemented for any hollow microstructure (e.g., nanostraw, nanotube, nanopore) due to a novel fabrication process that decouples the nanostructure from the underlying electrode.

## Results

### Fabrication of low-impedance nanovolcanoes

NVs have been previously employed for intracellular recordings of action potentials from primary cardiomyocyte cultures Desbiolles, de Coulon, Bertsch, Rohr, and Renaud^9^; Desbiolles, de Coulon, et al.^10^. However, these NVs are not amenable to seal resistance measurement because of their large impedance, as discussed in the supplementary materials (Supplementary Information, Section 1.1).

**Fig. 1:**
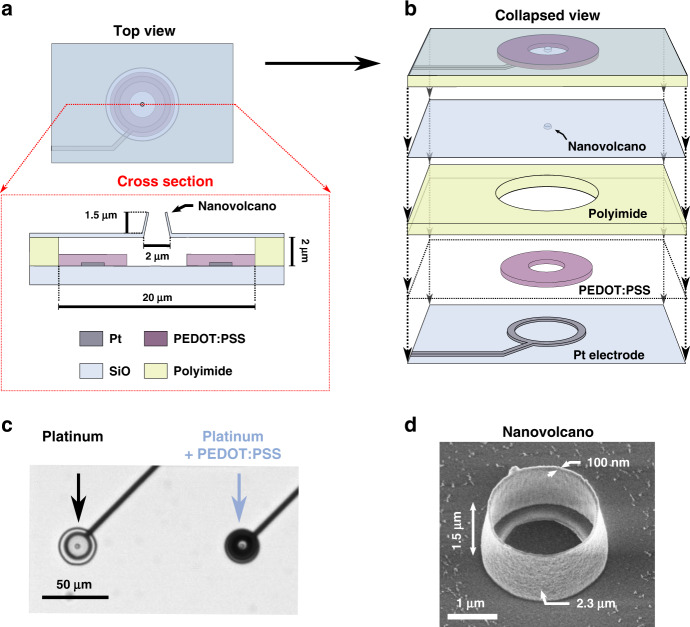
Device description. **a** Top view and cross section of the device to scale. The nanovolcano is standing on top of a cavity whose floor is patterned with a platinum electrode covered by a layer of poly(3-4,ethylenedioxythiophene) polymer doped with polystyrene- sulfonate (PEDOT:PSS). **b** Device collapsed view showing the different layers to scale. From bottom to top: platinum electrode on fused silica substrate, PEDOT:PSS ring, polyimide insulation with cavity etched into it and SiO_2_ insulating layer into which the nanovolcano is patterned. **c** Optical micrograph of the platinum electrode before and after in situ electrodeposition of PEDOT:PSS. Although the electrode is enlarged, the nanovolcano is still readily imageable thanks to the ring geometry and the transparent fused silica substrate. **d** Scanning electron micrographs of the finished device viewed with a tilt. Nanovolcanoes of various heights and diameters were fabricated in this study

We thus developed a new fabrication process to lower the impedance of NVs by over two orders of magnitude (for fabrication details, see Materials and Methods and Supplementary Fig. 3.1). A top view, cross-section and collapsed view of the finished device is depicted in Fig. 1a, b. The key feature of this device is its platinum ring electrode of 17 µm in diameter, covered by a layer of poly(3,4-ethylenedioxythiophene) polystyrene sulfonate (PEDOT:PSS). PEDOT:PSS is a conductive polymer characterized by a large specific capacitance that can be electrodeposited in situ and has long been used to lower the impedance of MEA Cui and Martin^11^.

The electrodeposition of PEDOT:PSS is performed as the last step of the fabrication process and is easily monitored under an optical microscope (Fig. 1c). Due to the ring-shaped platinum electrode, it is possible to stop the deposition process early to leave the portion of substrate below the NV transparent. This allows optical inspection of all NVs during cell culture to assess which NV should be considered for seal measurement (i.e., covered by a cell) and which should not (not covered).

**Fig. 2:**
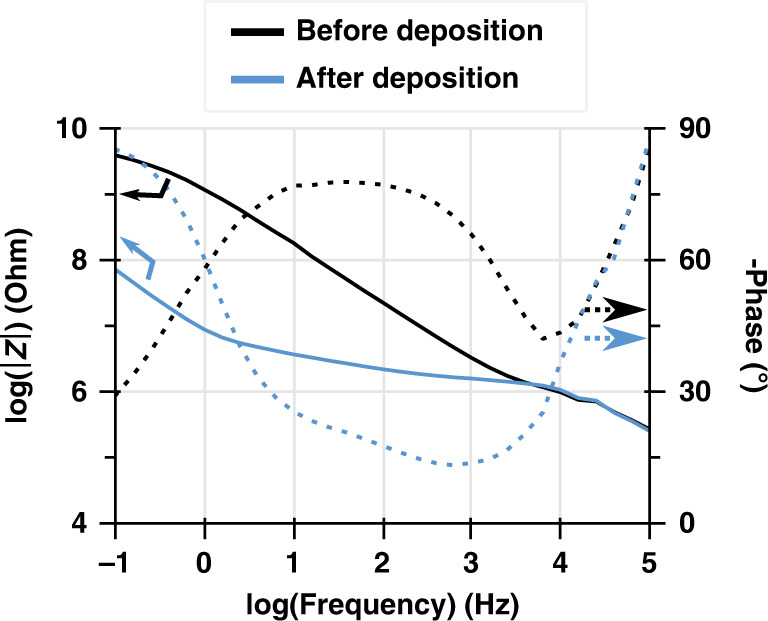
Bode plot of the magnitude and phase of the electrode impedance before and after electrodeposition of PEDOT:PSS. The impedance magnitude decreases significantly in the range of the seal resistance measurement between 1 Hz and 1 kHz. The impedance decreases above 10 kHz characterized by a −90° phase arises from the stray capacitance of the printed circuit board used to interface the devices. Between 10 kHz and 100 Hz, the impedance is dominated by the spreading resistance, between 100 and 10 Hz, a transition occurs from the spreading resistance to the PEDOT:PSS capacitance with a transitory regime characterized with a −45° phase arising from the diffusion of cations through the polymer (see Supplementary Fig. 3.3 for the equivalent circuit and theoretical model). The device under test had a layer of insulating polyimide 3 µm thick, one more micron than in the rest of the study, which results in smaller stray capacitance but has no other effect on the measurements

It should be noted that the NV itself is independent of the underlying electrode, a feature that we leverage below to alter the NV geometry (diameter and height; as highlighted in Fig. 1d) and obtain higher seal resistance while maintaining constant electrode impedance for direct comparisons. The electrodeposition process results in a decreased impedance below 10 kHz that reaches up to two decades (Fig. 2).

### Measurement modus operandi

The seal resistance of a cell adhered on top of a NV is measured by electrochemical impedance spectroscopy (EIS). In this study, we define seal resistance as the resistive element hindering current flow from the buried Pt/PEDOT:PSS electrode to the bath grounding electrode, particularly when a cell obstructs the NV. This resistive element arises from the electrolyte-filled cleft between the cell and its substrate. This definition of seal resistance is similar to its usual meaning in the context of electrophysiology recordings using MEA, although with our device, the cell is in contact with the NV, rather than the electrode itself. This setup is somewhat reminiscent of a traditional patch clamp recording where the actual electrode is not in direct contact with the cell and the seal resistance arises from the current leakage through the point of contact between the cell and rim of the patch pipette versus the grounded bath.

In a typical experiment, the cells are plated on a device housing an array of 60 NVs and allowed to adhere to the substrate for 24 h. During the measurement session, the device is connected to a potentiostat in a three electrode configuration (working electrode: NV’s Pt/PEDOT:PSS electrode, counter electrode: Pt wire and reference electrode: silver/silver-chloride (Ag/AgCl) pellet). From the impedance spectra, the seal resistance is determined as the averaged real part of the impedance over a decade centered at the point of minimum phase after subtraction of the same metric from a NV that is not covered by a cell. Alternatively, the impedance spectrum of NVs covered by a cell can be fitted to an equivalent circuit to extract the seal resistance. Both methods yielded similar results (Supplementary Fig. 3.4), so we used the former method since it allows for simpler data processing.

### Impact of chemical functionalization on the seal resistance

**Fig. 3:**
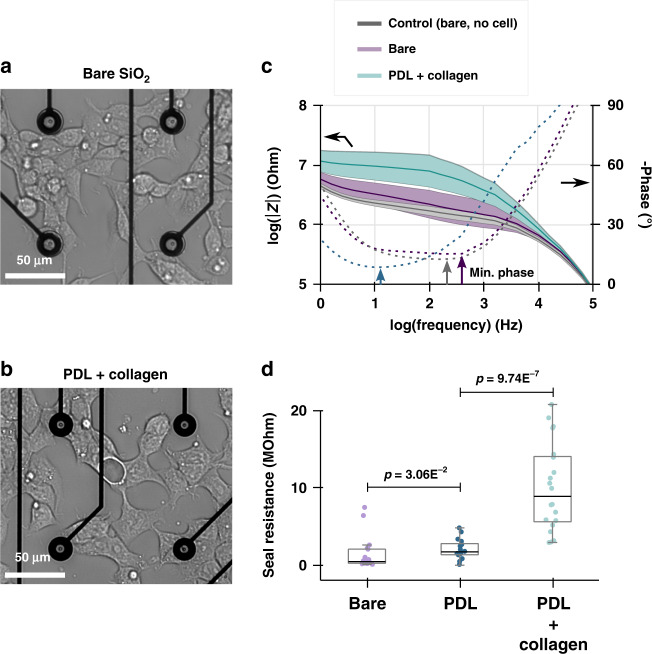
Impact of chemical functionalization on seal resistance. **a** Optical micrograph of human embryonic kidney cells (HEK) 24 h after plating on bare glass substrate shows elongated features with little spreading over the substrate. **b** In comparison, cells plated on glass substrate functionalized with poly-D-lysine (PDL) and collagen assume a flatter cell body configuration by spreading more on the substrate due to improved adhesion. **c** Impedance spectra displayed as a Bode plot of the magnitude (solid line) and phase (dashed line) for cells plated on bare and PDL + collagen-functionalized substrates. The impedance spectrum acquired from nanovolcanoes not covered by a cell is displayed as a control. The impedance magnitude is represented by the mean of the sample of cells interrogated ± one time the sample standard deviation represented by the colored area. Sealing resistances are obtained from the resistive portion of the spectra characterized by a minimum in the impedance phase (phase closest to 0° marked by colored arrows; see text for detail). **d** Comparison of seal resistances for samples of HEK cells cultured on bare glass (*n* = 17), PDL (*n* = 20) and PDL + collagen (*n* = 20). *p*-values correspond to a two-sided Mann‒Whitney *U*-test

As a demonstration of our method’s ability to sense the impact of chemical functionalization, we assessed the effect of different adhesion promoters on the seal resistance of human embryonic kidney cells (HEK) cultured on NVs. HEK cells were cultured on substrates that were either bare, functionalized with poly-D-lysine (PDL) alone, or PDL and collagen sequentially. It is apparent from optical inspection of cells cultured on bare or PDL/collagen functionalized chips that they adhere differently to the substrate, as evidenced by the difference in cell body shape (Fig. 3a, b). Qualitatively, this is also visible from the modulus of the impedance spectrum reaching larger values for HEK cells cultured with adhesion promoters (Fig. 3c). For quantitative analysis, the seal resistances are extracted and displayed as boxplots where each data point represents a measurement originating from a single cell (Fig. 3d).

From our data, we conclude that PDL results in a significantly better seal resistance compared to bare substrate (2.04 ± 1.2 vs. 1.45 ± 2.2 MΩ, respectively, mean ± std; two-sided Mann‒Whitney *U*-test, *p*-value = 3.06E^-2^). Similarly, PDL together with collagen resulted in significantly larger seal resistances compared to PDL alone (9.97 ± 5.7 vs. 2.04 ± 1.2 MΩ, respectively, mean ± std; two-sided Mann‒Whitney *U*-test, *p*-value = 9.74E^-7^). In these data, the spread of the distributions arises primarily from biological variability and random position of the cell over the NV, as opposed to variability due to noise in the measurement (Supplementary Fig. 3.5).

### Variation of the nanovolcano geometry

**Fig. 4:**
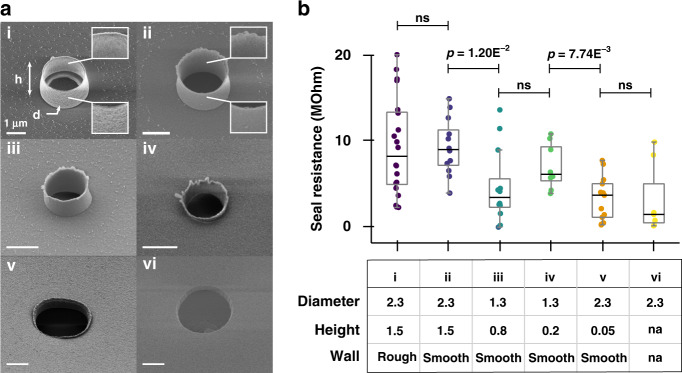
Impact of nanovolcano geometry on seal resistance. **a** Scanning electron micrograph of the different nanovolcano geometries fabricated. All scale bars correspond to 1 µm. Probe (i) and (ii) are 2.3 µm in diameter and 1.5 µm in height; however, (i) has walls with nanoscale roughness compared to (ii) smooth walls (insets). (iii) and (iv) are 1.3 µm in diameter but with decreasing wall height. (v) and (vi) are 2.3 µm in diameter with walls 50 nm high (v) or without walls (vi). For a summary of the geometrical parameters, see table in (**b**). where diameter and height are in units of microns. **b** Seal resistance of human embryonic kidney cells 24 h after plating on nanovolcanoes of different geometries. *p*-values correspond to a two-sided Mann‒Whitney *U*-test

Having demonstrated the ability of our method to discriminate changes in seal resistance, we altered the NV geometry to increase the cell/NV coupling. To do so, we fabricated NVs of varying diameter and height (Fig. 4a). We also fabricated NV of equal dimensions but with different roughnesses of the NV wall (rough vs. smooth; Fig. 4a, i vs. ii). Finally, we included chips with holes instead of NV (Fig. 4a, vi). After culturing HEK cells on all these NV geometries, we measured the seal resistance as described above and compared their distribution across the geometries (Fig. 4b). During experiments with HEK cells, we performed all measurements 24 h after plating cells at a low density of 31500 cells/cm^2^. We have characterized the impact of both plating density and time of recording and concluded that seal measurements should be compared at a similar time after plating, and that high plating densities reflect the properties of cell sheets rather than those of single cells (Supplementary Fig. 3.6 and 3.7).

Our data show that slight variations in the NV geometry can have a significant impact on the seal resistance; most notably, increasing the NV wall height from 50 to 200 nm (Fig. 4b) results in significantly higher seal resistances (6.93 ± 2.7 vs. 3.19 ± 2.6 MΩ, respectively, mean ± std; two-sided Mann‒Whitney *U*-test, *p*-value = 7.74E^-3^). Increasing the NV height further to 800 nm, however, resulted in a lower average seal resistance, although the difference was not statistically significant (5.04 ± 4.3 vs. 6.93 ± 2.7 MΩ, mean ± std; two-sided Mann‒Whitney *U*-test, *p*-value = 0.15). NVs with both a larger diameter (2.3 µm) and height (1.5 µm) resulted in larger seal resistances, although again this data showed no statistical significance (9.24 ± 3.3 vs. 6.93 ± 2.7 MΩ, mean ± std; two-sided Mann‒Whitney *U*-test, *p*-value = 0.13). The impact of the NV roughness was not found to significantly change for the height (1.5 µm) and diameter (2.3 µm) tested (9.97 ± 5.7 vs. 9.24 ± 3.3 MΩ rough vs. smooth wall, mean ± std; two-sided Mann‒Whitney *U*-test, *p*-value = 0.71).

### Electrophysiological recordings from primary cortex neurons

Having ascertained that our method can differentiate the impact of chemical and geometrical cues on cell seal resistance, we looked to apply it to optimize the coupling of primary rodent cortex neurons to NVs. We used NVs of different geometries as in our previous experiment (geometry i, ii, iii, iv, vi, Fig. 4a) to assess which geometry is preferable for electrophysiological recording from rodent cortex neurons. In all cases, the NVs were functionalized with PDL and laminin sequentially. The cells were allowed to mature for 16 days before performing the experiment, at which point each culture was inspected optically to identify which NVs were covered by cells. The impedance spectrum of each NV covered by a cell was acquired as described above, followed by recording of spontaneous electrophysiological activity. We expected that probe geometries/functionalization yielding large seal resistance would result in better electrophysiological recording. The quality of the electrophysiological signal was assessed by the signal-to-noise ratio (SNR) of action potentials measured extracellularly (EAP). Because intracellular access is often too low and yields too few data to enable a quantitative comparison, the extracellular signal was preferred. We report the distribution of mean EAP SNR as individual data points overlaid with boxplots that summarize the distribution data. Each data point therefore corresponds to a measurement originating from a single cell (i.e., average SNR of all the spikes recorded from the given cell).

**Fig. 5:**
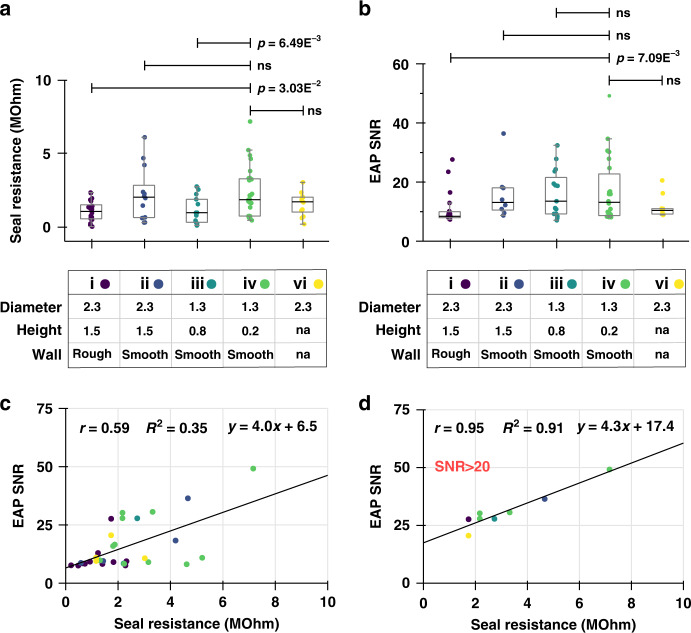
Seal resistance, extracellular action potential (EAP) signal-to-noise ratio (SNR) and their comparison for primary rodent cortex neurons after 16 days in culture. **a** Seal resistance of neurons on nanovolcanoes of different geometries. Groups (i–vi) are as in Fig. 4. **b** Average SNR of spontaneous EAP measured from neurons on nanovolcanoes of different geometries. In (**a**) and (**b**), each data point corresponds to a single cell, and the boxplots summarize the sample distribution for a given geometry. **c** Plot of the average EAP SNRs against seal resistances for all cells. The line is a linear regression characterized by the equation at the top of the plot, Pearson coefficient *r* and coefficient of determination *R*^2^. **d** Same as in (**c**) but only for cells whose average EAP SNRs were above 20. *p*-values correspond to a two-sided Mann‒Whitney *U*-test

In contrast to our results with HEK, primary rodent cortex neuron reached significantly larger seal resistances (Fig. 5a) with smaller NVs (2.30 ± 1.7 vs. 1.13 ± 0.9 vs. 1.03 ± 0.7 for geometries iv, iii and i, respectively, mean ± std; two-sided Mann‒Whitney *U*-test, *p*-values = 6.49E^-3^; 3.03E^-2^). On the other hand, the seal resistance of neurons on holes (no NV wall) was not found to be significantly different from that on NVs (1.53 ± 0.8 vs. 2.30 ± 1.7 for geometries VI and IV, respectively, mean ± std; two-sided Mann‒Whitney *U*-test, *p*-value = 0.25). The EAP SNR followed the same trend (Fig. 5b) with smaller NVs also yielding best results (29.78 ± 12.8 vs. 11.00 ± 1.4 for geometries iv and i respectively, mean ± sem; two-sided Mann-Whitney *U*-test, *p*-value = 7.09E^-3^).

The motivation of our study is that impedance spectroscopy predicts the quality of electrophysiological recording well and can thus be used as a substitute for time-consuming traditional electrophysiology measurements. When we plot the average SNRs against seal resistances, we observe a reasonably good fit to a linear regression defined by *SNR* = 4*R*_*seal*_ + 6.5, with R_seal_ expressed in MΩ (Fig. 5c). Under the hypothesis that NV recording with low EAP SNR might be occluded (i.e., separated from the spiking neuron by cell debris or a nonneuronal cell), we propose to consider only NVs with a minimum arbitrary average EAP SNR. For NVs recording a mean EAP SNR above 20, the correlation between impedance and electrophysiology data is markedly improved (Pearson coefficient = 0.95, coefficient of determination 0.91; Fig. 5d).

## Discussion

### Fabrication and characterization of low-impedance nanovolcanoes

The consistency of the deposition process was very satisfactory (Supplementary Fig. 3.2). Optical inspection during electrodeposition allowed us to leave the center of the ring electrode undeposited and thus allow it to remain optically transparent. The optical transparency of the cell/probe interface can allow multiplexed recordings such as electrical and fluorescence measurements for the development of voltage-sensitive dyes, recombinant proteins or optogenetic manipulation.

We analyzed the impedance spectrum of the Pt/PEDOT:PSS electrode following the model of Cui and Martin Cui and Martin^11^ for electrodeposited PEDOT:PSS films (Supplementary Fig. 3.3). In this model, the bulk electronic capacitance of the polymer is coupled (in series) with the diffusion of ionic charge carriers (cations) within the film and the spreading resistance of the electrode. The model fits the experimental impedance spectrum well considering the complexity of charge transfer within conducting polymers.

Based on the impedance spectrum after deposition and the noise level in our experiments, we estimate the lower and upper bound of seal resistance achievable with our approach respectively as 1 kΩ and 20 GΩ. These lower and upper bounds originate from the spreading resistance across the NV and our PCB stray impedance, respectively. The seal resistance of cardiomyocyte or neuron cells ranges from a few MΩ on flat substrates Braun and Fromherz^5^ to a few hundred MΩ on substrates with nanotopography Lin et al.^2^; R. Liu et al.^12^; Robinson et al.^3^. The dynamic range of our method is thus well suited to study adherent cell seal resistance. Additionally, the decreased impedance is expected to improve the SNR of electrophysiological recordings thanks to the lower thermal noise of the electrode.

### Impact of chemical functionalization on seal resistance

Seal resistance arises from the tortuous cleft between a cell’s plasma membrane and the substrate surrounding the electrode. It has long been known that chemical functionalization can alter the dimension of this cleft to values ranging from tens to a few hundreds of nanometers Zeck and Fromherz^13^. It is a logical outcome that a narrower cleft results in a larger seal resistance, and accordingly, the minimization of cell/substrate distance by chemical functionalization has been a common strategy to improve electrophysiological recordings from micro/nanoelectrodes.

An illustrative example of this approach is the internalization of micrometer-sized gold protrusions functionalized with RGD peptides by *Aplysia* neurons Spira et al.^14^. Such strategies have led to state-of-the-art intracellular electrophysiological measurements by MEA Hai, Shappir, and Spira^15^. Others have built upon these findings by modeling the penetration of the cell plasma membrane using vertical nanowires and concluded that adhesion forces are pivotal to enable intracellular access Xie et al.^16^. In this context, a method that would enable the assessment of chemical functionalization impact on seal resistance in a quantitative, high-throughput manner is highly desired.

The ability of chemical functionalization to modulate seal resistance is evident in our data showing an order of magnitude larger seal resistance when the appropriate adhesion promoter is used (Fig. 3c). This demonstrates the ability of our method to discriminate different patterns of cell adhesion. The typical values of seal resistance we measured from HEK cells on NVs are in close agreement with the 10 MΩ seal resistance reported for HEK cells cultured on planar substrate as assessed by fluorescence imaging of a voltage-sensitive fluorescent dye Braun and Fromherz^5^. However, others have reported seal resistances in the range of 100 to 500 MΩ for HEK cells on vertical nanowires with diameters of 150 nm and 3 µm in height Robinson et al.^3^. These data suggest that the NV does not raise the seal resistance significantly compared to a nanowire (in the case of HEK cells), which indicates another critical aspect of the cell/electrical probe interface requires consideration: the impact of the probe geometry and cell stiffness, as discussed below.

### Variation of the nanovolcano geometry

Previous electrophysiology recordings from cardiomyocytes using NVs have allowed for an estimation of the seal resistance as ~300 MΩ Desbiolles, de Coulon, et al.^10^, ~30 times larger than observed for the HEK cells in this study.

This discrepancy between cardiomyocytes and HEK cells highlights the importance of another factor on seal resistance: the interplay between the probe’s geometry and the stiffness of the cells. As discussed previously Xie et al.^16^, cells with stiffer plasma membranes (i.e., larger Young modulus) require taller nanostructures with a larger pitch to induce the same amount of membrane deformation as for a cell with a plasma membrane of lower Young modulus. Although the Young moduli of HEK and primary cardiomyocyte plasma membranes are unknown, we can consider a related parameter, the overall cell stiffness as calculated from indentation experiments, which suggests that HEK cells are stiffer than cardiomyocytes derived from human-induced pluripotent stem cells (5–7.5 kPa vs. 1 kPa, respectively) Pires, Shree, Manu, Guzniczak, and Otto^17^; Zhang, Keleshian, and Sachs^18^.

This comparison supports our observations of lower seal resistance for HEK cells compared to cardiomyocytes when cultured on NVs. Previous impedance measurements with NVs fabricated on an AFM tip are also in agreement with this conclusion, as the impedance of a NV engaged on adherent cells resulted in a larger impedance increase for cardiomyocytes than for HEK cells Desbiolles, Hannebelle, et al.^19^. Since in that study the NV was brought into contact with the cell, the contribution of adhesion forces can be ruled out, and hence, the difference in seal resistance must arise from the physicochemical properties of the plasma membrane being different for different cell types.

While the physicochemical properties of the cell’s plasma membrane are not easily within control of the investigator, the geometry and pitch of the microstructures interfacing the cells are. To obtain a high-quality electrophysiology recording, an investigator may accordingly refine the design of the recording micro/nanoelectrode to find the best parameters (e.g., height, diameter, pitch), as evidenced by a large seal resistance. Our experiment with NVs of different geometries demonstrates such a framework wherein taller NV walls result in larger seal resistances (Fig. 4b). It is particularly interesting that increasing the NV height alone eventually led to a decrease in seal resistance, whereas increasing height with a proportional increase in diameter resulted in a larger mean seal resistance. This observation may suggest that the NV diameter affects the cell/microstructure interface in a similar way as the pitch of a nanowire array does, i.e., by raising the topographic prominence of the NV wall. While the different geometries of NV investigated thus far have resulted in a modest increase in the seal resistance for HEK cells, this experiment successfully demonstrated the ability of impedance spectroscopy to resolve the impact of microstructure geometry on seal resistance. In the future, taller NVs with larger diameters seem to be a promising design to interface HEK cells, which is interesting considering the extensive use of engineered spontaneously spiking HEK cells for in vitro electrophysiology Dipalo et al.^20^; Park et al.^21^.

### Electrophysiology recordings from primary cortex neurons

A noteworthy feature of the extracellular recordings by this new generation of NVs is that they mostly recorded signals from only one cell (sometimes two, but never more). This situation is reminiscent of small electrodes of subcellular size that interface with single cells. With such electrodes, however, the SNRs of the EAPs are typically decreased due to a larger electrode impedance Viswam, Obien, Franke, Frey, and Hierlemann^22^, which was not the case with our large, buried electrodes. Furthermore, a given EAP was always registered by one electrode only (Supplementary Fig. 3.8), which is expected for arrays with large electrode-to-electrode pitch. These combined features made the analysis of EAP trivial, with spike sorting only needed for a few channels and facilitated by having at most two candidates during classification (Supp. information Section 1.6 and Supplementary Fig. 3.9).

In this experiment, we found that NVs with smooth walls and shorter height and diameter are preferred to interface primary mammalian neurons (Fig. 5a, b). While the mean EAP SNR for that specific geometry was twice as high as for any other geometry investigated, the improvement is only statistically significant with respect to the original, larger NV design. We believe that the large variability in these data originates from the random positioning of the cell over the NV. For a cell well centered over a nanostructure and completely covering it, adhesion forces are expected to decrease the cell/substrate distance, leading to higher seal resistance. On the other hand, the case of a cell off-center or partially covering a nanostructure may result in asymmetric loading of the plasma membrane and failure to induce membrane tension and deformation. According to this hypothesis, a strategy for optimal positioning of the cell over the nanostructure would drastically reduce the variability in seal resistance and EAP SNR.

Another interesting outcome of this experiment was the very robust correlation of seal resistance to EAP SNR for NVs registering strong electrophysiology activity, with rather poor correlation demonstrated for cells registering low SNR electrophysiological signal (SNR above and below 20, respectively, Fig. 5c, d). It was particularly surprising to find a few examples of NVs covered by cells with large seal resistances but poor EAP SNRs. This observation could be explained by some NVs being obstructed by cell debris while being simultaneously covered by an active neuron. The presence of dead, yet substrate-adhering cell bodies/debris was routinely observed in our culture due to our use of frozen neurons, which result in ~50% cell death after thawing and plating. In such cases, the seal resistance may be higher due to this debris, while the EAP SNR would be decreased by the increased spreading resistance between the electrode and the live cell.

It is also possible that our culture contained both neurons and nonneuronal cells. 2D mixed neural cell cultures are often found to organize as carpets of glial cells on top of which neurons reside Limongi et al.^23^, which would result in the same effect over seal resistance and EAP SNR as for cell debris. In our experiments, we attempted to limit the presence of glial cells by using only prenatal neurons from the cortex of embryos sacrificed at the 18^th^ day of development, at which time differentiation of neural progenitors to glial cells has yet to begin Bandeira, Lent, and Herculano-Houzel^24^. Based on these two plausible explanations, it could be argued that only NVs registering EAP with SNRs above a certain threshold truly represent our scenario of interest: a neuron in direct contact with a NV. This hypothesis should be further ascertained with live/dead staining and immunostaining of glial and neuronal markers (e.g., glial fibrillary acidic protein and microtubule-associated protein 2, respectively).

The seal resistances found in experiments with primary rodent neurons were between 6 kΩ and 10 MΩ, a range that is much lower than the hundreds of MΩ reported for nanowires R. Liu et al.^12^; Robinson et al.^3^. We conclude that the NV geometries investigated thus far are not optimal in addressing mammalian neurons. However, the method presented here allowed us to resolve seal resistance in a manner that was well correlated with electrophysiological recording quality, which is the main object of this study and should allow for incremental optimization procedures. In conjunction with the development of a cell positioning strategy (e.g., dielectrophoresis Zhou et al.^25^, chemical patterning X. Liu et al.^26^ or nanotopography Huang, Delacruz, Ruelas, Rathore, and Lindau^27^, the process of optimizing micro/nanoelectrodes for intracellular electrophysiology will progress toward rational design frameworks).

## Conclusion

In this study, we presented a novel approach for the characterization of the interface between cells and electrical probes. We demonstrated that the measurement of the seal resistance through impedance spectroscopy captures the degree of coupling between the cell and nanostructure well. Furthermore, this measurement correlates well with the quality of electrophysiological recording. The measurement is able to resolve physiologically relevant patterns in cell/substrate adhesion even for very low values of seal resistance (down to a few kΩs).

The data obtained show the critical role of chemical functionalization in the establishment of a tight cell/probe interface. However, the impact of the various probe geometries investigated so far resulted in lesser modulations of the seal resistance. Taken together, these observations suggest that adhesion processes are more potent than topography-induced membrane deformation, although a more exhaustive range of probe geometries should be considered to ascertain this conclusion.

From a broader perspective, the new generation of NV described offered better quality of electrophysiological recordings in terms of noise and SNR due to their decreased impedance. Furthermore, we discovered that the new NV configuration with the electrode buried within the insulating substrate results in electrophysiological recordings that are fairly robust to background activity originating from distant neurons. Together with the possibility of measuring access resistance after permeabilization of the cell membrane (Supplementary Information, Section 1.3), this truly single-cell framework could foster the development of new bidirectional measurement methods. With such methods, the coupling of the cell to the underlying electrode could be assessed in terms of seal and access resistance during concomitant electrophysiological recording. These readouts could be used to (i) maintain a consistent access resistance (e.g., by applying electroporation when the access resistance falls below a set threshold) and (ii) correct the registered signal for signal attenuation at the cell/electrode interface on a cell-to-cell basis.

A critical advantage of the decoupled electrode and nanostructure configuration rational used in this study is the resulting ability to selectively alter the nanostructure over an arbitrary range of dimensions, without affecting the electrode properties. This feature is broadly appealing since other nanostructures developed by other investigators (e.g., nanotube, nanostraw, nanopore) could be fabricated on top of the buried electrode in a straightforward fashion.

Finally, we suggest that this measurement modality is not constrained to the characterization/optimization of probes dedicated to electrophysiology but is also appealing for the development of new cell adhesion factors, fundamental investigations of cellular mechano-sensing and toxicology studies.

## Materials and methods

### Fabrication of the device

The stepwise fabrication process flow is depicted in the supplementary materials (Supplementary Fig. 3.1). Before starting the process, fused silica substrates are cleaned in two consecutive piranha baths (three parts sulfuric acid 97% and one Part 30% hydrogen peroxide) for 5 min each, followed by thorough rinsing in two consecutive ultra-pure deionized water (DIW) baths before spin-drying. In step (A) a stack of thin metal layers consisting of Ti/Pt with thickness 10/240 nm is evaporated using an EVA760 (Alliance Concept, France) e-beam evaporator. In step (B), the ring electrodes, leads and contact pads are patterned in the metal layers. This is achieved by spin-coating the substrate with a 600 nm thick AZ ECI 3007 i-line photoresist (MicroChemicals, Germany) with an ACS Gen 3 automated spin-coater (Su¨ss MicroTec, Germany). The desired pattern is then exposed using a MA6GEN3 mask aligner (Su¨ss MicroTec, Germany) in i-line mode (365 nm) with a dose of 165 mJ/cm^2^ and developed with the same automated coater starting with a 60 s postexposure bake with a proximity of 100 µm to a hotplate set to 110 °C, cooling for 15 s on a cool plate and subsequent development in AZ MIF726 developer with a total contact time of 27 s. Before etching, the resist is reflown by direct contact with a Sawatec HP200 hot plate (Sawatec, Switzerland) set to 125 °C for 60 s. The metal layer is then etched using Ar^+^ ion bombardment using an IBE350 (Veeco, USA) set to 500 V acceleration voltage, 800 mA beam current and with a stage tilt with respect to the incident beam of −30°. The stage tilt during etching is used to limit redeposition of the etched material on the photoresist sidewalls, which would result in fences. The etching is monitored using an integrated secondary ion mass spectrometer (SIMS) and stopped 10 s after the appearance of the silicon signal originating from etching of the fused silica substrate. The photoresist is then removed thoroughly by subjecting the substrate to long oxygen plasma etching at 500 W with an oxygen flow of 400 mL/min for 7 min using a TePla 300 microwave plasma system (PVA TePla, USA). When stripping the resist in this manner, the wafers were positioned vertically using a quartz holder. In step (C), a 2 µm thick layer of polyimide is spin coated and cured before plasma enhanced chemical vapor deposition (PECVD) of a layer of silicon carbide and silicon dioxide. Before polyimide coating, the substrates were dehydrated for 10 min in a convection oven set at 150 °C. A quick oxygen plasma with the same parameters as in step (A) was applied to remove any organic contaminants. The substrate was then immediately spin coated (WS-650, Laurell Technologies, USA) by manual dispensing of 3 mL over static substrate with a solution of aminopropyl triethoxysilane silane (VM-652 adhesion promoter; HD Microsystems, USA) before spinning for 30 s at 3000 RPM under a nitrogen stream. PI2610 polyimide (HD Microsystems, USA) was spin coated (LSM-200; Sawatech, Switzerland) at 3000 RPM for 40 s to obtain a final thickness (after curing and hard bake) of 2 µm. After spin coating, the polyimide was cured by direct contact of the substrates with a hotplate (HP-401Z, Sawatech, Switzerland) at 65 °C for 3 min and 105 °C for 3 min. The polyimide was hard baked in a convection oven (T6060; Heraeus, Germany) for 1 h at 300 °C under a nitrogen atmosphere above 200 °C. Before the PECVD deposition, the substrates were exposed to a mild oxygen plasma of 100 W with an oxygen flow of 400 mL/min for 1 min to roughen the polyimide surface to improve adhesion. Robust adhesion of the silicon-based dielectric to polyimide is critical to the final device stability, especially for experiments involving week-long cell cultures in aqueous saline conditions. A thin adhesion layer of silicon carbide was thus deposited (35 nm; chamber pressure of 1000 mTorr, gaz flow of 750 sccm of 2% SiH4 in Ar and 70 sccm of CH4, 20 Watts RF) before silicon dioxide (320 nm; chamber pressure of 1000 mTorr, gaz flow of 400 sccm of 2% SiH4 in N2 and 710 sccm of N2O, 20 Watts RF) using an Oxford Plasmalab System 100 (Oxford Instruments, UK) with the deposition chamber temperature set to 300 °C. In step (D), the NVs are defined as described previously Desbiolles et al.^9^. A 1.6 µm thick layer of AznLoF2020 photoresist (MicroChemicals, Germany) is spin coated and 2.25 µm diameter openings are defined by exposure with a VPG200 direct laser writer (Heidelberg, Germany) with a 355 nm UV light dose ranging from 9 to 15 mJ/cm^2^ depending on the nanovolcano geometry. To obtain a photoresist straight wall, the manufacturer’s exposure dose recommendation for a 1.6 µm thick layer of AznLoF2020 is 80 mJ/cm^2^, but we found that optimal values to obtain the desired pattern lateral dimension were significantly different on the glass substrate and dependent on the pattern dimension. Therefore, the exposure dose used in this step should be optimized on a substrate/design basis. The postexposure bake is conducted at 110 °C with a 100 µm proximity gap for 75 s followed by 51 s contact time development with AZ MIF726. The substrates are then subjected to ion beam etching as described above with a 0° incidence angle. Etching was stopped 2 min after the disappearance of the silicon signal on the SIMS detector. The photoresist is removed, and the cavity below the NV is formed with oxygen plasma as described in step (B) except that the wafer was lying flat in the chamber (for better homogeneity) and that the etching time was prolonged for a total duration of 20 min or until the under-etching of the polyimide reached a diameter of 20 µm. SEM images were acquired using a Merlin SEM (Zeiss, Germany) with an extraction voltage of 1.5 kV and a beam current of 30 pA and a secondary-electron detector. The wafers were diced on a DAD321 (Disco, Germany) with a resinoid blade 70 µm in width under 25000 RPM rotation moving at 1 mm/s from the top side. of the wafer. A glass O-ring was glued on top of the individual chips using PDMS and cured overnight at 60 °C in a convection oven.

### Electrodeposition of PEDOT:PSS

Electrodeposition was performed from a filtered aqueous solution of 20 mM 3,4-ethylene dioxythiophene (EDOT, 483028; Merck, USA) and 1 wt % poly(sodium 4-styrenesulfonate) with a molecular weight of 70,000 g/mol (PSS, 243051; Merck). The electrodeposition was performed according to a previously demonstrated protocol Rothe, Frey, Madangopal, Rickus, and Hierlemann^28^. The devices were first cleaned with a 3 min oxygen plasma (100 W, 650 mTorr; Diener Electronic, Germany) followed by immediate filling of the culture chamber with pure ethanol to ensure proper wetting of the inside of the NVs. The culture chamber content was then exchanged with deionized water (DIW) 6 times, making sure to leave a thin layer of water each time to keep the cavity below the NV filled with liquid. DIW was then exchanged two times with the EDOT:PSS solution, and the device was connected to a Stat.h bipotentiostat (Ivium, The Netherlands) in a three electrode configuration.

We used a silver-silver chloride electrode in saturated NaCl (MF-2052; BASI, USA) as a reference electrode and a platinum wire as a counter electrode. Electrodeposition was achieved by chronoamperometry, alternating between 0.23 V and 1.05 V for 800 ms and 200 ms, respectively, and the total number of cycles was between 400 and 600 depending on the NV geometry. The process was monitored under an optical microscope and stopped when the ring electrode enlargement left a transparent circular surface area with a diameter of 7 µm. The culture chamber content was finally exchanged with deionized water (DIW) six times.

### Cell culture

During all liquid exchange of the cell culture chamber, a thin layer of liquid was always left to prevent the cavities below the NVs from filling with air. In some cases, the devices were functionalized with adhesion promoters before cell patting. When PDL (A-003-E; Merck) was used as an aqueous solution of 500 µg/mL was incubated over the device at room temperature (RT) for 2 h before rinsing with DIW 3 times and cell plating. If and additional adhesion promoter was used, PDL functionalization was carried out on the day before plating in the same way, followed by overnight incubation at 4 °C with either collagen reconstituted in aqueous acetic acid 2% (v/v) at 100 µg/mL. (11179179001; Roche, Switzerland) or laminin at 100 µg/mL in PBS (10010023; ThermoFisher, USA). In both cases, the adhesion promoter solutions werewashed off with three rinses with PBS before cell plating. HEK cells (CRL- 1573) were obtained from ATCC. All experiments with HEK cells corresponded to culture between passages 5 and 15. HEK cells were kept in DMEM supplemented with Glutamax (10566016, ThermoFisher), 10% fetal bovine serum (F9665, Merck) and 0.4% penicillin/streptomycin (P4333, Merck) solution within a 37 °C incubator under a 5% CO_2_ and 100% humidity atmosphere. Before plating, an 80% confluent culture was collected by trypsinization (1084440001, Merck) for 5 min at 37 °C followed by mechanical dislodgement by repetitive pipette dispensing over the cells. Cells were centrifuged for 2 min at 0.3 RCF and resuspended in culture medium after the supernatant was discarded. We plated 30000 cells per chip (0.95 cm^2^) and conducted Experiments 24 h after passaging unless stated otherwise. Primary rat cortex neurons from Day 18 embryos (A36511, ThermoFisher) were frozen, thawed for 2 min in a 37 °C water bath and diluted slowly in neurobasal cell culture media (A3582901, ThermoFisher) to avoid osmotic shock. We found that centrifugation of the cell solution to remove the freezing solvent resulted in higher viability; thus, the cells were spun at 0.3 RCF for 2 min. The supernatant was discarded, and the cells were resuspended in neurobasal medium supplemented with 100X Glutamax (35050061, ThermoFisher), B27 supplement (A3582801, ThermoFisher) and 0.4% penicillin/streptomycin (P4333, Merck). A total of 200 000 cells were plated per chip (0.28 cm^2^) unless specified otherwise. Half of the culture medium was exchanged every 2nd day, and experiments were performed 16 days after plating.

### Electrochemical impedance spectroscopy and seal resistance measurement analysis

Electrochemical impedance spectroscopy (EIS) was performed with a Stat.h bipotentiostat (Ivium, The Netherlands) in a three electrode configuration. We used a silver-silver chloride pellet (E205; Multichannel Systems, Germany) as a reference electrode and a platinum wire as a counter electrode. The impedance spectrum was acquired from 200 kHz to 1 Hz (100 mHz for characterization of the electrode) under a sinusoidal excitation waveform of 10 mVrms and a 0 V DC bias. The application of a fixed DC bias rather than the open circuit potential is preferable, as different levels of conductive polymer oxidation can result in different bulk capacitances. The measurement was performed within a Faraday cage to limit electromagnetic noise interference. From the impedance spectrum, the seal resistance was extracted from one of the two following approaches. The seal resistance was obtained by finding the point of minimum phase across the spectrum. For the NV presented in this study, this corresponded to a typical frequency between 10 Hz and 1 kHz. The real part of the impedance was averaged over a decade around that frequency, and this metric was subtracted from the same value obtained from NVs not covered by any cell to obtain the seal resistance. Alternatively, an equivalent lumped element circuit model was fitted to the experimental impedance spectrum from which the seal resistance was obtained. Both approaches are depicted and compared in the supplementary information Supplementary Fig. 3.4.

The distributions of seal resistance are summarized and reported as the mean ± one time the standard deviation in the main text. The distribution of seal resistance data often deviated significantly from normality (Shapiro‒Wilk test, significance level α = 0.05). Accordingly, we used a nonparametric test to test for significant differences across experimental conditions. We employed two-sided Mann‒Whitney *U*-test for which the null hypothesis was that the data obtained from the two experimental conditions come from the same distribution. The null hypothesis was rejected for *p*-values below the significance level α = 0.05.

### Electrophysiology recordings and analysis

Before recordings, the cell culture medium was exchanged for recording buffer (in mM; 125 NaCl, 5.5 KCl, 1.8 CaCl_2_, 0.8 MgCl_2_, 20 HEPES, 24 glucose, and 36 mOsm sucrose at pH 7.3, osmolarity adjusted to 315 mOsm with sucrose). The device was interfaced with a custom PCB and placed within an incubator set to a temperature of 37 °C. CO_2_ perfusion was not necessary with our recording solution buffered with HEPES. The recording amplifier used was a HS-36 headstage (1 TΩ, 2 pF input impedance) connected to a Digital Lynx SX acquisition system (Neuralynx, USA). The signal ground of the amplifier was connected to a silver-silver chloride pellet immersed in the cell culture bath, and the chassi ground of the amplifier was connected to the inner metal lining of the incubator. Spontaneous neuronal activity was sampled at 32 kHz over a range of ±10 mV. The signal was bandpass filtered between 1 and 5000 Hz for visualization and 300 and 3000 Hz for spike analysis. Spikes were extracted from the filtered signal given that their maximum absolute voltage reached was greater than 2.3 times the value of the median absolute deviation for that particular channel.

In most recordings, the median absolute deviation was below 10 µV. The SNR was calculated as the absolute ratio of spike peak voltage to the value of the median mean deviation for that channel. The spike SNRs were averaged cellwise; hence, each data point in Fig. 5c corresponds to the average EAP SNR of a single cell. In the main text, the distribution of the mean EAP SNR is thus reported as the mean ± one times the standard error of the mean.

## Supplementary information

Supplementary_materials

## Data Availability

The data supporting this manuscript are available upon reasonable request.
