# Supplementary material for: Impedance spectroscopy of the cell/nanovolcano interface enables optimization for electrophysiology
Source: Microsyst Nanoeng. 2023 May 16;9:62. doi: 10.1038/s41378-023-00533-z (PMC10188357; doi:10.1038/s41378-023-00533-z)
Supplement: Supplementary file 1 — Supplementary_materials [file 41378_2023_533_MOESM1_ESM.pdf]

Impedance spectroscopy of the cell/nanovolcano interface enables optimization for electrophysiology

Supplementary materials

Nicolas Maïno[1*], Arnaud Bertsch[1] and Philippe Renaud[1]

[1]Microsystems laboratory 4, Institute of Electrical and Micro Engineering, Ecole Polytechique Fédérale de Lausanne (EPFL), Lausanne, Switzerland, Rte Cantonale, Lausanne, 1015, Vaud, Switzerland.

*Corresponding author(s). E-mail(s): nicolas.maino@epfl.ch; Contributing authors: arnaud.bertsch@epfl.ch; philippe.renaud@epfl.ch;

1 Supplementary Information **3**
1.1 Comparison to previous nanovolcanoes generation 3
1.2 Comparison of nanovolcanoes noise levels 3
1.3 Opportunity for access resistance measurements 4
1.4 Equivalent circuit model and defining equations of the electrode and nanovolcano 4
1.5 Equivalent circuit model and defining equations of the cell-covered electrode and nanovolcano 6
1.6 Analysis of channels registering action potentials from more than one cell 6

2 References **8**

3 Supplementary Figures **10**
3.1 Fabrication process flow of the low impedance nanovolcanoes . 10
3.2 Homogeneity of the PEDOT:PSS electrodeposition 11
3.3 Equivalent circuit of the buried PEDOT:PSS electrode 12
3.4 Estimation and fitting of the seal resistance 13
3.5 Noise in the impedance spectroscopy measurements 14
3.6 Effect of plating density on HEK cells seal resistance 15
3.7 Effect of day post plating on HEK cells seal resistance measurement 16
3.8 Independence of recording channels during electrophysiology . . 17
3.9 Analysis of channels with more than one unit 18
3.10 Averaged waveforms of spontaneous extracellular action potentials 19
3.11 Impact of chemical functionalization on primary neurons seal resistance 20
3.12 Comparison of the previous and new generation of NV for seal resistance measurement 21
3.13 Inferred noise of the previous and new generation of NV 22
3.14 Feasibility of access resistance measurement by impedance spectroscopy 22

1 Supplementary Information

1.1 Comparison to previous nanovolcanoes generation

The capability of resolving difference of seal resistance through impedance spectroscopy as presented in this study was modeled with an equivalent circuit of the cell/NV interface. Specifically, we looked at the ability of the new and former NVs ability to i) resolve minute change of the seal resistance and ii) the dependence of the measured signal on properties of the cell membrane in contact with the NV. We first conclude that although theoretically possible, the measurement of seal resistance with the previous implementation of NV necessitates rigorous fitting given that the impedance spectrum shape changes markedly for modest increase in seal (Supp. Figure 3.12). This is in opposition to the new NV presented in this study whose impedance spectrum keeps the same shape while being merely scaled up by increasing sealing resistance allowing for easy and unambiguous assessment. Still, the application of the previous generation of NV to seal measurement is further impaired by the impact of the nearby cell membrane capacitance. Because of the large impedance of the previous NV, the measurement would need to be taken at higher frequencies (10-100 kHz). Although the capacitance of the portion of cell membrane in contact/close proximity of the electrode is very small (in the range of fraction of picofarads), the use of such high frequencies results in shunting of the seal resistance by capacitive current through the cell. This poses a significant problem considering that the impedance of this portion of membrane may vary from cell to cell and for different experimental conditions thereby resulting in the same sealing resistance yielding a different impedance spectrum. In comparison, seal measurements with the NVs presented in this study are insensitive to the nearby cell membrane impedance owing to the decreased electrode that allows measurements over lower frequencies range (typically 0.01-1 kHz).

1.2 Comparison of nanovolcanoes noise levels

We compared the performance of the new NV with respect to their original implementation in terms of impedance and noise. The noise amplitude spectral density (ASD) was inferred from the thermal noise of NVs using the real part of the impedance spectrum according to the following formula: $R_{thermal} = \sqrt{4kT\Re(Z_{elec})f}$ (Supp. Figure 3.13). The root-mean-square value of the noise in specific bandwidths relevant to electrophysiology reveals that the NV presented in this study are superior. The NV presented in this study register 1.03 µVrms compared to 9.88 µVrms for the previous NV implementation in the 1-300 Hz bandwidth relevant to slow electrophysiological processes (*e.g.*, synaptic inputs, membrane oscillation) and 8.72 µVrms and 12.06 µVrms over the 0.3-5 kHz bandwidth relevant to action potentials.

1.3 Opportunity for access resistance measurements

In the comparison above, we concluded that the NVs presented in this study measure seal resistance with little interference from the junctional cell membrane capacitance. Interestingly, it follows that since little capacitive current will flow through an intact cell membrane there should be a large, easily readable change after permeabilization of the membrane; for example with electroporation. Since most probes described in the literature relies on some form of membrane perforation technique (*e.g.*, electro- Lin, Xie, Osakada, Cui, and Cui (2014); Robinson et al. (2012), opto- poration Dipalo et al. (2017)) of the cell membrane to establish intracellular contact, it would be highly beneficial to be able to measure the resistance of the patch of membrane itself (*i.e.*, access resistance). Since the flow of current through the cell is necessarily shunted by the seal resistance, it follows that access resistance can only be measured given that it is on the same order of magnitude as the seal resistance. Values reported for the junctional cell membrane resistance, the portion of cell membrane interface by the probe, are typically in the order of 100 GΩ Ojovan et al. (2015) for an intact membrane and a few 100s of MΩ for an electroporated membrane Lin et al. (2014); Robinson et al. (2012). According to our simulations based on the equivalent circuit depicted in Supp. Figure 3.14, it is reasonable that access resistance of a few GΩ can be resolved with the NV described in this study. This could open up a promising bi-directional framework where the access resistance is controlled, *e.g.*, by re-applying electroporation when its value falls below a certain set point due to membrane resealing, meanwhile an intracellular electrophysiological signal is recorded and corrected for the amplitude drop/temporal deformation resulting from access resistance and cell/probe interface characteristics.

1.4 Equivalent circuit model and defining equations of the electrode and nanovolcano

The equivalent circuit model is characterized by the following elements. The finite-length Warburg element represents the diffusion resistance of cations through the polymer film according to equation 1:

$$Z_{warburg} = \frac{(\frac{\tau_D}{C_D})coth(j\omega\tau_D)^{1/2}}{(j\omega\tau_D)^{1/2}} \tag{1}$$

Where D is the diffusional time constant, C_D the diffusional pseudocapacitance. The ratio of τ_D/C_D defines a diffusional resistance R_D associated with cations diffusion from the polymer/electrolyte interface into/out of the polymer film as the polymer changes its oxidation (doping) state. This impedance element is named differently (finite space Warburg, open Warburg element, finite-length Warburg with reflective boundary) in different research articles/-textbook/fitting software. Here we consider the case of a diffusional impedance arising from the diffusion of cations through a nanoporous material (*i.e.*, the

polymer layer) that is terminated by a reflective boundary (*i.e.*, the noble metal electrode). This gives rise to the *coth* function which replaces the *tanh* function used in the case of a transmissive boundary. Equation 1 is also often expressed in terms of admittance Y_0 which is the admittance at an angular frequency of 1 (rad/s) and a characteristic time $B = \frac{\delta}{\sqrt{D}}$.

The constant phase element is defined by equation 2):

$$Z_{CPE} = \frac{1}{C_b (j\omega)^n} \tag{2}$$

Where C_b is the bulk electronic capacitance (unit of $\mathrm{s^n}/\Omega$ hence not a capacitance in the traditional sense) and n is the phase constant parameter. In their modeling of electrodeposited PEDOT:PSS film, Cui and Martin (2003) used a capacitor instead of a phase element as we did here. The phase angle of our Pt/PEDOT:PSS electrodes impedance at low frequencies deviated significantly from that of a pure capacitor and we found that a constant phase element resulted in significantly better fit. Although the fitting of the low frequency end of the impedance spectrum had little effect on the accuracy of the spreading resistance fitting (the parameter of interest), we report here on the improved agreement of our model and experimental data using a constant phase element. We hypothesize that this deviation from the model of Cui and Martin (2003) may arise from different packing levels of the polymer originating from different electrodeposition conditions. Constant phase elements are traditionally associated with roughness or fractality of the electrode as proposed by Mulder, Sluyters, Pajkossy, and Nyikos (1990). Another possible explanation could be thickness or composition variation of the polymer film as discussed by Schiller and Strunz (2001)

The impedance associated with C_{stray} is defined by equation 3:

$$Z_{stray} = \frac{1}{j\omega C_{stray}} \tag{3}$$

The stray capacitance was dominated by our PCB stray capacitance since the stray capacitance of the electrodes leads on the chip was only 0.24 pF. When performing electrophysiology measurement we used a different high impedance PCB to prevent signal attenuation from the stray capacitance Desbiolles et al. (2020).

Hence the total impedance is as in equation 4):

$$Z_{total} = \frac{Z_{stray}(Z_w + Z_{CPE} + Z_{spread})}{Z_{stray} + Z_w + Z_{CPE} + Z_{spread}} \tag{4}$$

1.5 Equivalent circuit model and defining equations of the cell-covered electrode and nanovolcano

The equivalent circuit model is characterized by the following elements. The junctional and non-junctional cell impedance are defined following equation 5 and 6 respectively:

$$Z_{junctional} = \frac{R_j(1/j\omega C_j)}{R_j + (1/j\omega C_j)} \tag{5}$$

$$Z_{non-junctional} = \frac{R_{nj}(1/j\omega C_{nj})}{R_{nj} + (1/j\omega C_{nj})} \tag{6}$$

R_{nj} , C_{nj} , R_j and C_j were taken as 100 MΩ, 30 pF Robinson et al. (2012), 100 GΩ and 0.1 pF (from a specific capacitance of 1 µF/cm^2 and surface area of 12.6 µm^2 corresponding to the NV inner surface area). All other components are as in supplementary Section 1.4 and Figure 3.3. The new total impedance is given by equation 7:

$$Z_{total} = \frac{Z_{stray}(Z_w + Z_{CPE} + R_{spread}\frac{R_{seal}(Z_j+Z_{nj})}{R_{seal}+Z_j+Z_{nj}})}{Z_{stray} + Z_w + Z_{CPE} + R_{spread}\frac{R_{seal}(Z_j+Z_{nj})}{R_{seal}+Z_j+Z_{nj}}} \tag{7}$$

1.6 Analysis of channels registering action potentials from more than one cell

Most electrodes in our data registered extracellular action potentials (EAP) from only one cell. In some rare cases some channels picked up signals from two different cells. The process of classifying EAPs as originating from different cells is called spike sorting and is a well studied problem that grows increasingly difficult with the number of units (*i.e.*, firing cells) registered by a single electrode. In our recordings, classification of channels detecting several units was however relatively easy because those channels picked up spikes from at most two cells. Such a channel is displayed in supplementary Figure 3.9. Spike sorting was achieved with a minimalistic strategy that was yet good enough to cluster spikes belonging to different units effectively. The collection of spikes from the considered channel was normalized and subjected to principal component analysis. Classification was performed by k-means clustering by taking only the first two principal components and the number of clusters was adjusted manually by the user. Although one of the cells in the data presented in supp. Figure 3.9 is bursting frequently, the family of classified spikes show a satisfactory homogeneity. We believe the reason that we rarely observe channels with two units and no channels with three or more units is a consequence of the burrowed electrode configuration of our multi-electrode array (MEA). In a traditional planar MEA, the electrodes are on the same plane as the cells and typically register spikes originating from many units.

It is known that smaller electrodes will pick up signals from fewer units than larger electrode which makes analysis easier and less error-prone but comes with the trade-off of electrode with higher impedance and lower SNR Harris, Quiroga, Freeman, and Smith (2016); Viswam, Obien, Franke, Frey, and Hierlemann (2019). The NV implementation presented in this study has the merit of having low impedance thanks to the large ring electrode surface area while maintaining a small footprint on the substrate plane resulting on interfacing by mostly one and at most two cells.

2 References

References

Cui, X., & Martin, D.C. (2003, March). Electrochemical deposition and characterization of poly(3,4-ethylenedioxythiophene) on neural microelectrode arrays. *Sensors and Actuators B: Chemical*, *89*(1), 92–102.

10.1016/S0925-4005(02)00448-3

Desbiolles, B.X.E., de Coulon, E., Maïno, N., Bertsch, A., Rohr, S., Renaud, P. (2020, August). Nanovolcano microelectrode arrays: Toward long-term on-demand registration of transmembrane action potentials by controlled electroporation. *Microsystems & Nanoengineering*, *6*(1), 1–12.

10.1038/s41378-020-0178-7

Dipalo, M., Amin, H., Lovato, L., Moia, F., Caprettini, V., Messina, G.C., ... De Angelis, F. (2017, June). Intracellular and Extracellular Recording of Spontaneous Action Potentials in Mammalian Neurons and Cardiac Cells with 3D Plasmonic Nanoelectrodes. *Nano Letters*, *17*(6), 3932–3939.

10.1021/acs.nanolett.7b01523

Harris, K.D., Quiroga, R.Q., Freeman, J., Smith, S.L. (2016, September). Improving data quality in neuronal population recordings. *Nature Neuroscience*, *19*(9), 1165–1174.

10.1038/nn.4365

Lin, Z.C., Xie, C., Osakada, Y., Cui, Y., Cui, B. (2014, February). Iridium oxide nanotube electrodes for sensitive and prolonged intracellular measurement of action potentials. *Nature Communications*, *5*(1), 3206.

10.1038/ncomms4206

Mulder, W.H., Sluyters, J.H., Pajkossy, T., Nyikos, L. (1990, June). Tafel current at fractal electrodes: Connection with admittance spectra. *Journal of Electroanalytical Chemistry and Interfacial Electrochemistry*, *285*(1), 103–115.

10.1016/0022-0728(90)87113-X

Ojovan, S.M., Rabieh, N., Shmoel, N., Erez, H., Maydan, E., Cohen, A., Spira, M.E. (2015, September). A feasibility study of multi-site,intracellular recordings from mammalian neurons by extracellular gold mushroom-shaped microelectrodes. *Scientific Reports*, *5*(1), 14100.

10.1038/srep14100

Pouzat, C., Delescluse, M., Viot, P., Diebolt, J. (2004, June). Improved Spike-Sorting By Modeling Firing Statistics and Burst-Dependent Spike Amplitude Attenuation: A Markov Chain Monte Carlo Approach. *Journal of Neurophysiology*, *91*(6), 2910–2928.

10.1152/jn.00227.2003

Robinson, J.T., Jorgolli, M., Shalek, A.K., Yoon, M.-H., Gertner, R.S., Park, H. (2012, March). Vertical nanowire electrode arrays as a scalable platform for intracellular interfacing to neuronal circuits. *Nature Nanotechnology*, *7*(3), 180–184.

10.1038/nnano.2011.249

Schiller, C.A., & Strunz, W. (2001, August). The evaluation of experimental dielectric data of barrier coatings by means of different models. *Electrochimica Acta*, *46*(24), 3619–3625.

10.1016/S0013-4686(01)00644-2

Viswam, V., Obien, M.E.J., Franke, F., Frey, U., Hierlemann, A. (2019). Optimal Electrode Size for Multi-Scale Extracellular-Potential Recording From Neuronal Assemblies. *Frontiers in Neuroscience*, *13*.

3 Supplementary Figures

3.1 Fabrication process flow of the low impedance nanovolcanoes

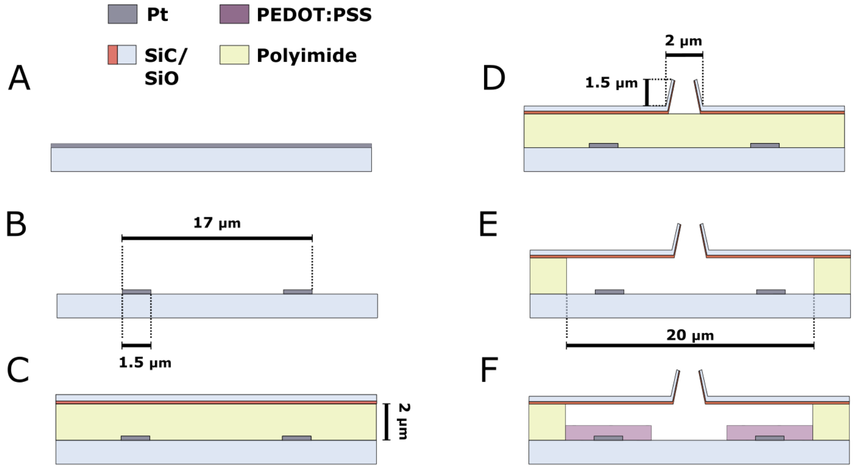

Fig. 3.1 Nanovolcano fabrication process flow. See Materials and Methods for detailed description (A) Metal evaporation, (B) electrode and leads patterning, (C) polyimide spin-coating and curing followed by plasma enhanced chemical vapor deposition of silicon carbide + silicon dioxide, (D) patterning of nanovolcanoes and access holes to contact pads, (E) cavity etching by isotropic oxygen plasma, (F) electrodeposition of PEDOT:PSS.

3.2 Homogeneity of the PEDOT:PSS electrodeposition

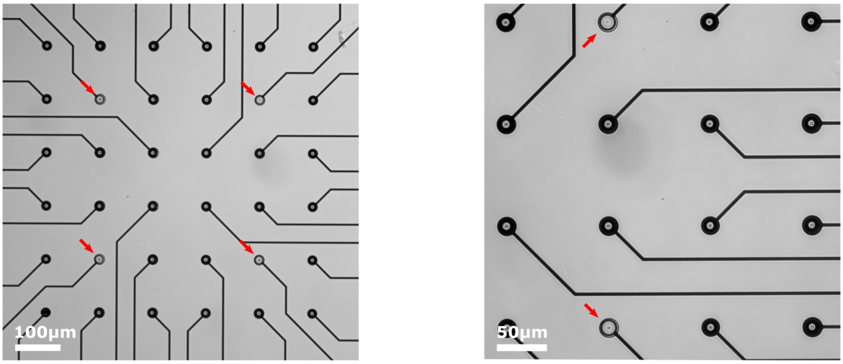

Fig. 3.2 Optical micrograph of a portion of the multi-electrode array after electrodeposition of PEDOT:PSS. On the left and right image the red arrows indicate electrodes that were voluntarily not deposited for comparison. During electrodeposition the ring shape of the electrode is seen to enlarge progressively until it covers most of the cavity as best visible on the magnified image (right). As can be seen the deposition homogeneity was very satisfactory with all 56 (60-4) electrodes of the array covered in a similar way.

3.3 Equivalent circuit of the buried PEDOT:PSS electrode

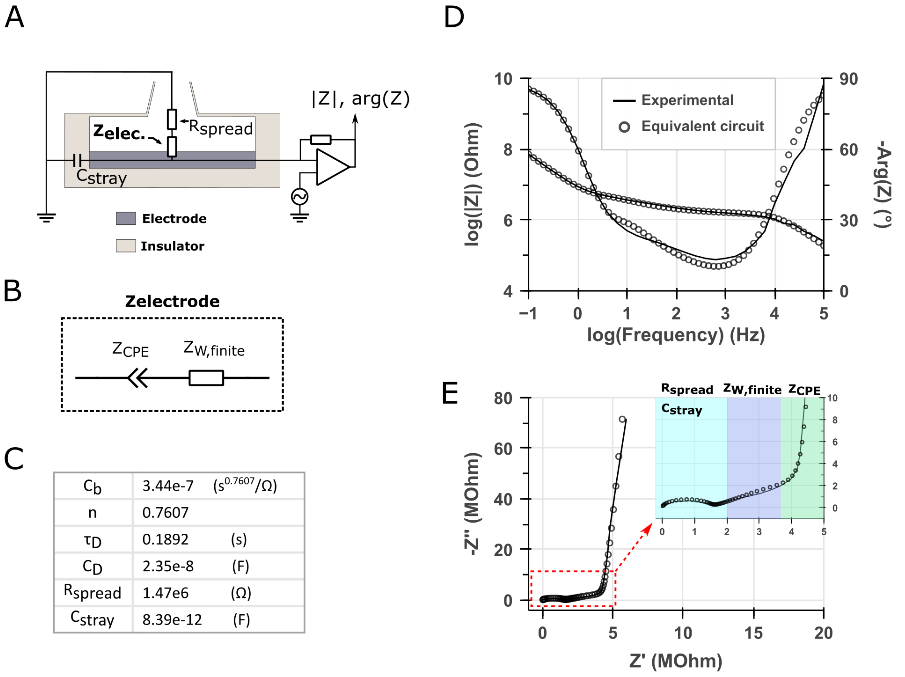

Fig. 3.3 Equivalent circuit of the electrode/NV and modeling of the experimental impedance spectrum. (A) The electrode/NV was modeled as a two branch circuit composed of i) the electrode impedance $Z_{electrode}$ in series with the spreading resistance R_{spread} which is in parallel with ii) the stray capacitance of the electrode leads and printed circuit board tracks C_{stray} . (B) $Z_{electrode}$ the electrode impedance is modeled according to Cui and Martin (2003) as a finite-length Warburg element in series with a constant phase element. The equations for each element and the total impedance are given above in the Supplementary information Section 1.4. (C) Fitted parameter using the model in (A). (D) Fitting the equivalent circuit model to the experimental results shows a good agreement of our model as evidenced by the almost overlapping curve on the Bode and (E) Nyquist plots. A deviation was often apparent around 1 Hz which arises from the bulk capacitance rising faster than the finite Warburg impedance. In (E) the inset highlights the different regime of the impedance spectrum and the corresponding dominating circuit element over that frequency range.

3.4 Estimation and fitting of the seal resistance

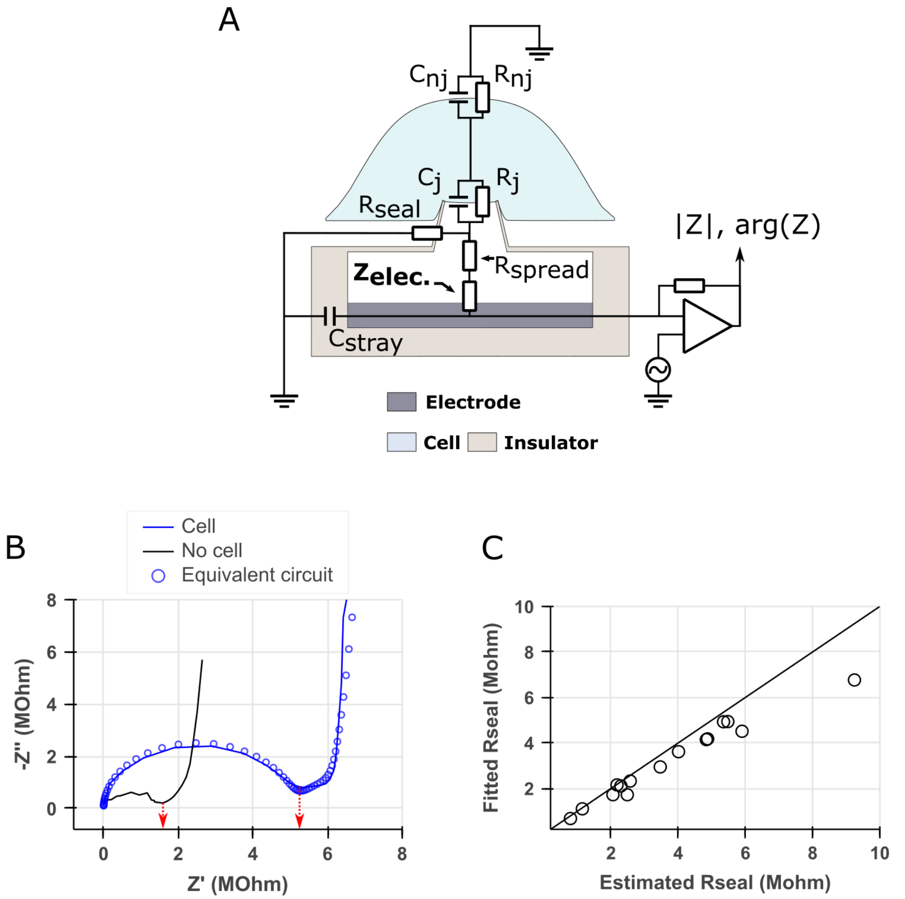

Fig. 3.4 Estimation and fitting of the seal resistance. (A) An equivalent circuit model of the cell on top of the nanovolcano can be fitted to the experimental data to extract the sealing resistance. (B) Alternatively, seal resistance can be estimated from the impedance spectrum without fitting. The impedance spectra of two NVs are displayed on a Nyquist plot. One NV is covered by a HEK cell (confirmed by optical inspection). The difference across the spectra arises from the seal resistance added by the cell. The seal resistance can be estimated reliably by taking the difference of the projection of the spectra on the real axis at the point of minimum phase (highlighted by red arrows). (C) Comparison of the two methods. Seal resistances fitted or estimated as in (A) or (B) are similar as evidenced by a coefficient of determination $R^2 = 0.97$.

3.5 Noise in the impedance spectroscopy measurements

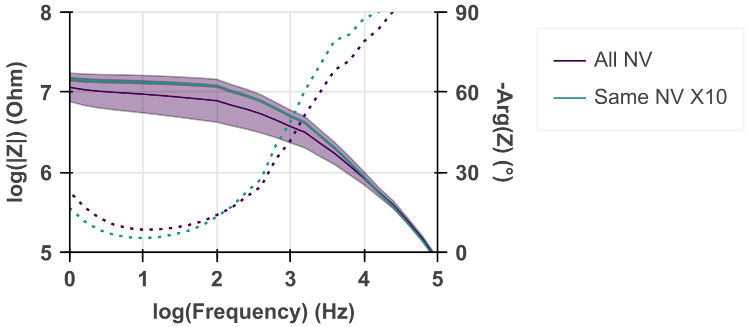

Fig. 3.5 Noise in the measurement in comparison to biological variability of the data. The distribution of impedance spectra for human embryonic kidney cells (HEK) on NV is displayed as a bode plot in magnitude (solid line) and phase (dashed line). The distribution is displayed as a colored band of ± one time the sample standard deviation centered around the mean. In the legend, “All NV” refers to the distribution of spectra obtained from n=20 HEK cells whereas the “Same NV X10” corresponds to a single HEK cell whose impedance spectrum was measured ten times in a row. Accordingly, the precision of the measurement was good enough that the variability in samples of many cells arises from the distribution of seal resistance rather than noise.

3.6 Effect of plating density on HEK cells seal resistance

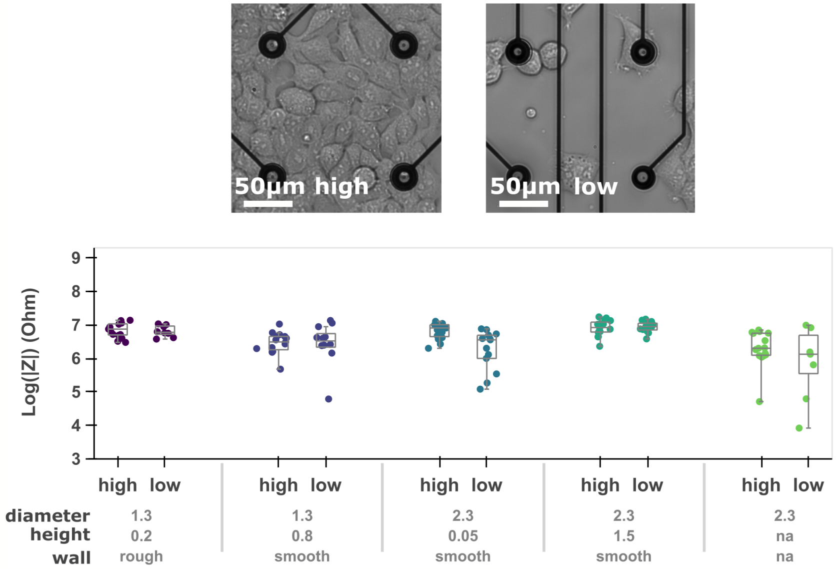

Fig. 3.6 Effect of plating density on seal resistance measurement. Human embryonic kidney cells (HEK) were plated at different densities on NV of different geometries and allowed to attach to the substrate for 24h before measurement of the seal resistance. Although the seal resistances were rather similar in both cases, we noted a tendency for the seal resistance to be higher when measurement from dense cultures. We hypothesize that this arises from cells forming a continuous monolayer at higher densities in which case the seal resistance measured might represent the sealing resistance of several cells in series.

3.7 Effect of day post plating on HEK cells seal resistance measurement

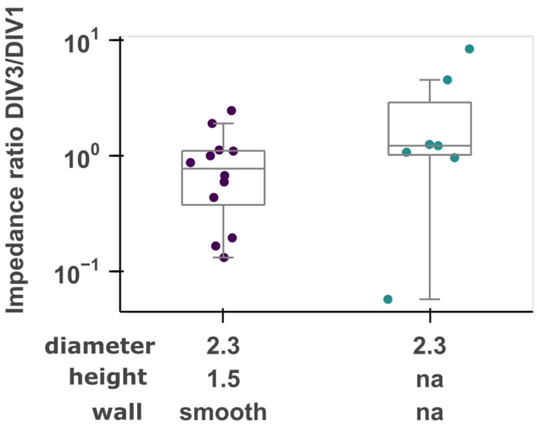

Fig. 3.7 Effect of day post plating on seal measurement. HEK cells were plated on NV of different geometries and their sealing resistance was measured after 24h or 72h. The seal resistance of the same cells was found to vary by as much as 20 fold between those two time points. In consequence only seal measurements carried out in the same half-day were compared to one another in this study. This also highlights the need for a harmless, longitudinal measurement method as the value seal resistance is fluctuating along the culture lifespan.

3.8 Independence of recording channels during electrophysiology

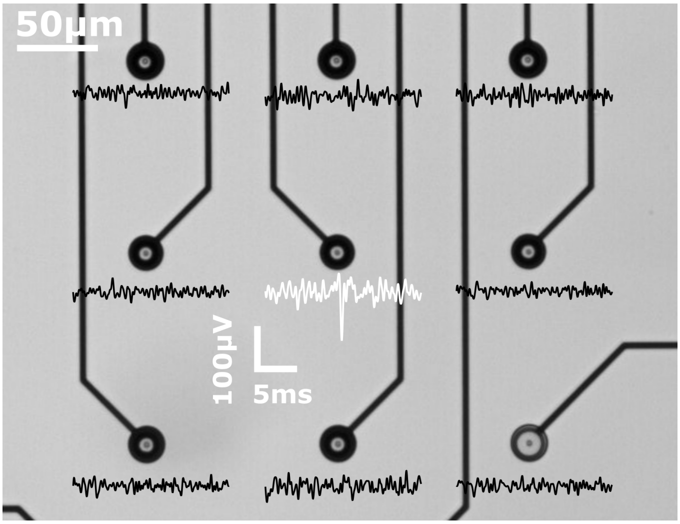

Fig. 3.8 Parallel electrophysiological recordings of neighboring electrodes displayed on a dummy optical image of the electrode array. The recordings were obtained from a culture of primary rodent cortex neurons 14 days after plating. All signal traces correspond to the same time window. An extracellular action potential is registered on the center electrode but is not detected on neighboring electrodes. This is expected for multi-electrode arrays of pitch equal and above 100 µm Harris et al. (2016) which makes analysis much easier given that action potentials emitted by a given cell are picked up by only one electrode at most.

3.9 Analysis of channels with more than one unit

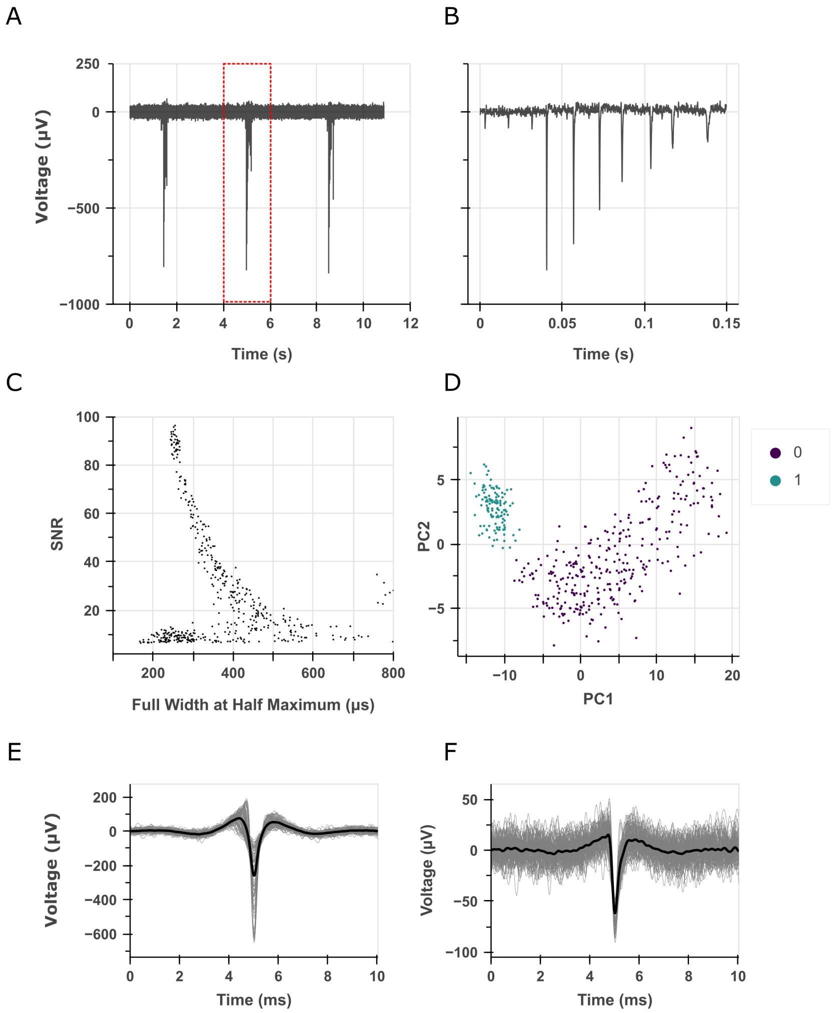

Fig. 3.9 Analysis of channels with more than one unit (A) Electrophysiological trace showing extracellular action potentials (EAP) originating from two different cells, one of which is bursting frequently. (B) Magnified view of the dashed red box in (A). Spikes of less than 100 μV in amplitude from one cell are visible prior to the burst followed by the burst itself originating from another cell emitting several action potentials in a row of decreasing amplitude. Bursting cells can be difficult to analyze as the decreasing amplitude of the spikes can lead to misclassification and are typically met with complex analysis methods Pouzat, Delescluse, Viot, and Diebolt (2004). In our data however, classification is easily achieved based on the large dissimilarity of the waveforms. (C) This dissimilarity is already obvious in the two dimensional space of spike SNR versus full width at half maximum. (D) Following a simple analysis (see supplementary information section 1.6), spikes are readily classified by k-means clustering. (E) and (F) the corresponding classified waveforms show good intra-class homogeneity.

3.10 Averaged waveforms of spontaneous extracellular action potentials

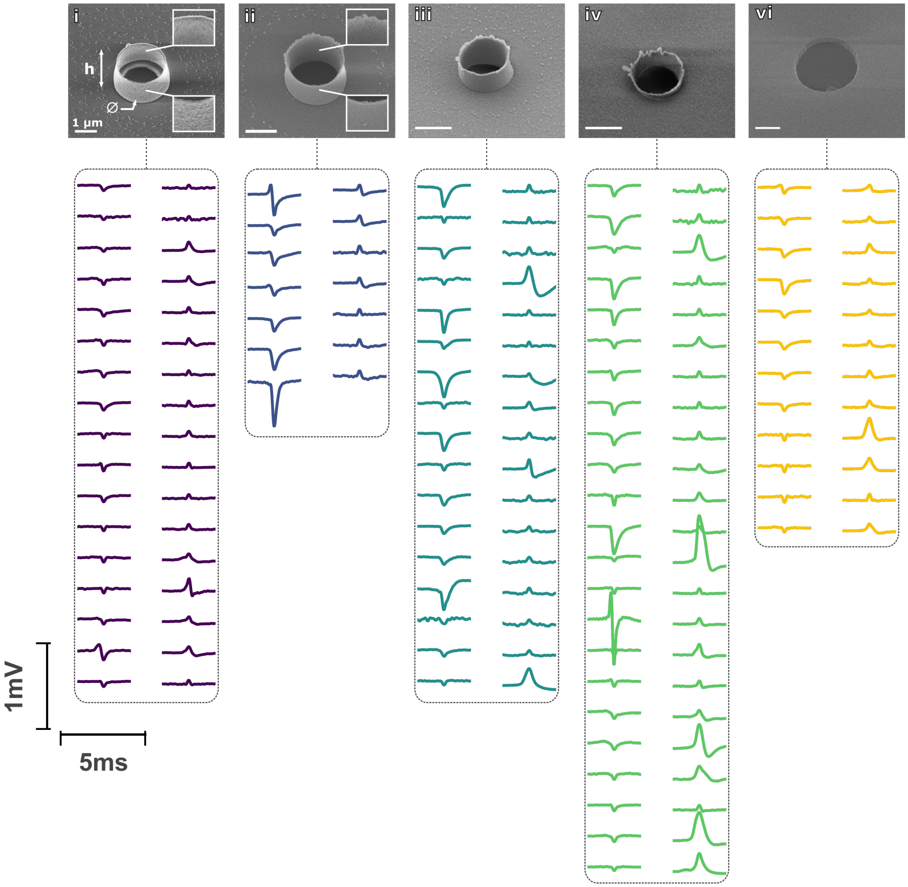

Fig. 3.10 Averaged waveforms of spontaneous extracellular action potentials (EAP) registered at nanovolcanoes of different geometries from primary rat cortex neurons culture 16 days after plating. The curves are offset in time and voltage for clarity and the color groups correspond to NV of geometries i, ii, iii, iv and vi as depicted by the SEM images on top of Figure 4. Each waveform was obtained from a single nanovolcano and were either monophasic hyperpolarization corresponding to EAP generated close to the soma, biphasic swings originating from axons and monophasic depolarization tentatively corresponding to attenuated intracellular action potentials.

3.11 Impact of chemical functionalization on primary neurons seal resistance

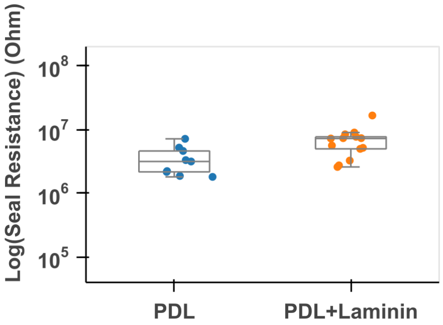

Fig. 3.11 Impact of chemical functionalization on seal resistance for primary rat cortex neurons culture 16 days after plating. Neurons were plated on NV functionalized with either poly-D-Lysine (PDL) alone or with PDL and laminin sequentially. Neurons on PDL and laminin had a 2.3 times larger seal resistance compared to those plated on PDL alone (7.24 MΩ and 3.15 MΩ respectively).

3.12 Comparison of the previous and new generation of NV for seal resistance measurement

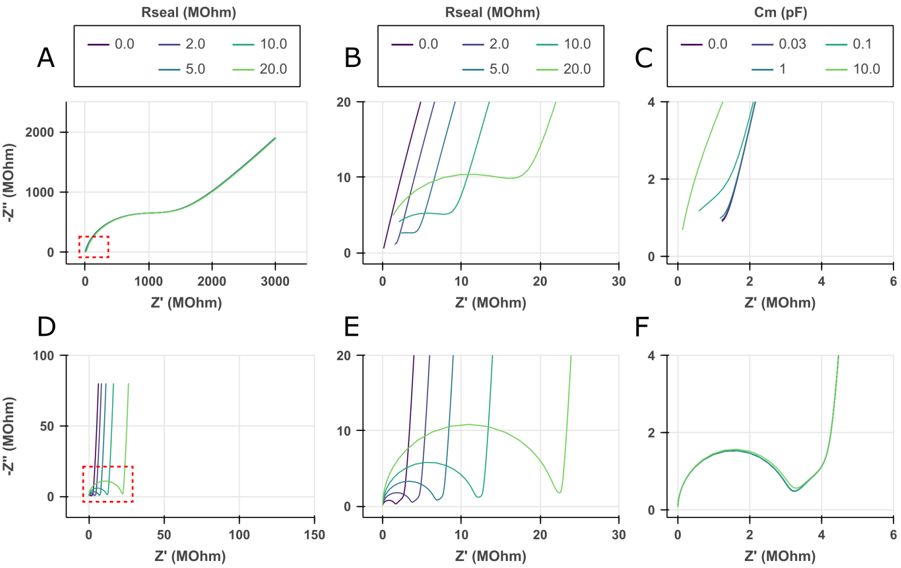

Fig. 3.12 Comparison of the previous generation of NV Desbiolles et al. (2020) to the new NVs presented in this study for the measurement of seal resistance. (A) and (D) are simulated impedance spectra displayed on a Nyquist plot for increasing value of seal resistance. (A) Corresponds to the measurement performed with the previous generation of NV and (D) to the one presented in this study. The legend in (A) also apply for (D). The model used in this simulation is as in supplementary Figure 3.4. (B) and (E) are magnification of the dashed red box in (A) and (D) respectively. (C) and (F) are the impedance spectra simulated for a constant seal resistance of 1.5 MΩ but with varying junctional cell membrane capacitance for the previous and new generation of NV respectively. In the former case (C), the impedance spectrum is largely dependant on the exact value of the junctional membrane capacitance in the range of 30 fF (plasma membrane sitting flat on top of the NV), 100 fF (plasma membrane protruding inside the NV), 1 pF (partially porated plasma membrane by *e.g.*, electroporation) and 10 pF (perfectly disrupted plasma membrane; the resulting capacitance then corresponds to the non-junctional membrane capacitance). On the other hand, the spectrum simulated for the new generation of NV (F) is independent of the capacitance of the cell junctional membrane.

3.13 Inferred noise of the previous and new generation of NV

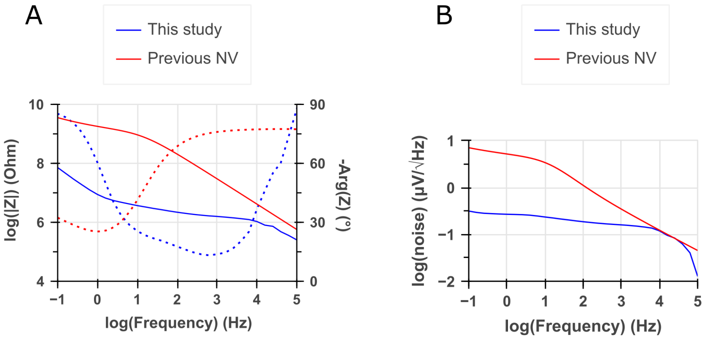

Fig. 3.13 Comparison of the impedance spectrum and inferred noise of the previous generation of NV Desbiolles et al. (2020) with respect to the new NVs presented in this study. The inferred noise was obtained from the electrodes impedance spectrum as described in the supplementary information section 1.2.

3.14 Feasibility of access resistance measurement by impedance spectroscopy

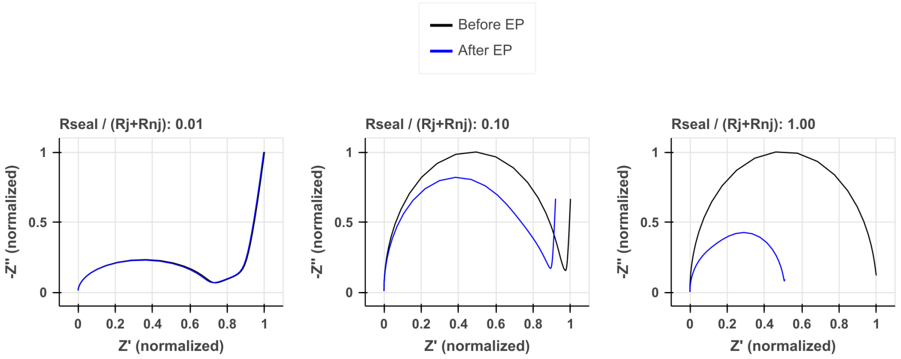

Fig. 3.14 Simulation of the impedance spectrum before and after electroporation for increasing value of $\frac{R_{seal}}{R_j+R_{nj}}$. The model used in this simulation is as in supplementary Figure 3.4. The black and blue curves (before/after electroporation respectively) correspond to a junctional membrane resistance of 100 GΩ and 100 MΩ respectively with the later being a typical value reached after electroporation Lin et al. (2014). For a seal resistance no less than one tenth that of the sum of junctional and non-junctional membrane resistance, the access resistance could be estimated from the decrease in the real part of the impedance at the point of minimum phase and a theoretical value of R_{nj}.
